# Surgical Obliteration of a Filum Terminale Arteriovenous Fistula Following Aborted Endovascular Embolization: Illustrative Case Report and Literature Review

**DOI:** 10.7759/cureus.75083

**Published:** 2024-12-04

**Authors:** Jordyn Mullins, Drew A Thibault, Alejandro Pando, Priyank Khandelwal, Ali T Meybodi, Amit Singla

**Affiliations:** 1 Department of Neurological Surgery, Burrell College of Osteopathic Medicine, Las Cruces, USA; 2 Department of Anatomical Sciences, Liberty University College of Osteopathic Medicine, Lynchburg, USA; 3 Department of Neurological Surgery, Rutgers University New Jersey Medical School, Newark, USA

**Keywords:** arterio-venous fistula, microsurgery, myelopathy, spinal cord edema, spinal cord venous congestion

## Abstract

Arteriovenous fistulas of the filum terminale are rare vascular malformations that predominantly affect males and can present with various neurological symptoms. In this study, we indexed previously published cases of filum terminale arteriovenous fistulas demonstrating that endovascular and microsurgical management are both proven to be appropriate and successful treatment modalities with low complication rates. Endovascular treatment is far less invasive; however, it is associated with higher failure rates, which need to be managed surgically. In this case, we report a 64-year-old male patient who presented with lower back pain and bilateral lower extremity weakness. He was found to have a filum terminal arteriovenous fistula causing thoracolumbar spinal cord edema. Following a failed attempt of endovascular embolization complicated by declining neuromonitoring signals, open microsurgical obliteration of the lesion was successfully performed. While endovascular management of filum terminale arteriovenous fistulas is a viable and successful treatment modality in select cases, surgeons should be prepared to manage these cases with an open microsurgical approach should embolization fail or become unsafe. Proper radiologic characterization of the lesion and accurate localization of the location of the fistula are requisites to a safe and successful obliteration of these lesions.

## Introduction

Arteriovenous fistulas (AVFs) of the filum terminale (FTAVFs) are rare vascular malformations that can present with symptoms ranging from low back pain (LBP) to severe radiculopathy [1]. Overall, vascular malformations of the spine are relatively rare (3% of all spinal arteriovenous shunts), with lesions occurring caudal to the conus medullaris infrequently observed [2]. FTAVFs are perimedullary arteriovenous malformations (AVMs) that are found on the surface of the pia mater and are without a capillary bed between arterial and venous systems [3]. These lesions are classified as type IV arteriovenous malformations of the spinal cord and are subcategorized into type IVa, type IVb, and type IVc by Anson and Spetzler [4]. Type IVa lesions are low-flow AVFs supplied by a single anterior spinal artery (ASA) branch. Type IVb lesions are intermediate-flow fistulas with multiple arterial feeders. Type IVc lesions are high-flow fistulas supplied by several ASA or posterior spinal artery branches [1,5]. Over time, these fistulas contribute to the development of myelopathic or radicular symptoms, secondary to abnormal vascular flow and venous congestion, resulting in arterial insufficiency [6,7]. Treatment for FTAVFs includes endovascular embolization or open microsurgical resection [2]. The treatment choice is made for each patient individually, depending on vascular characteristics and institutional resources [2]. Importantly, lesions that are not completely obliterated surgically or endovascularly are at high risk of recurring with worsening of symptoms. In this report, we present the case of a 64-year-old male who presented to the hospital with lower back pain and proximal bilateral lower extremity weakness. Additionally, we provide a current literature review of reported cases of FTAVFs.

## Case presentation

**Figure 1:**
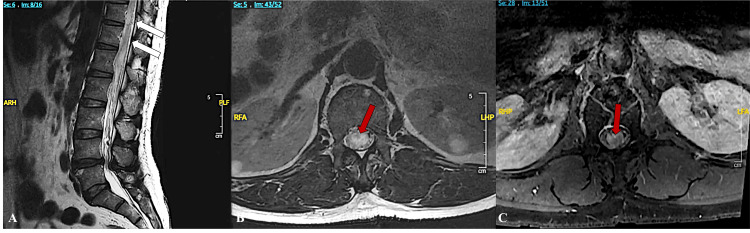
Preoperative magnetic resonance imaging (MRI). Preoperative sagittal T2 sequence MRI (A) exhibited thoracolumbar spinal cord signal change consistent with spinal cord edema. T2 sagittal MRI imaging demonstrates intradural vessels overlying the spinal cord (white arrows). Axial T2 (B) and T1 (C) sequence views at the L1 vertebral level also show central cord signal change (red arrows). Images (A) and (B) have been lightened for clarity.

**Figure 2:**
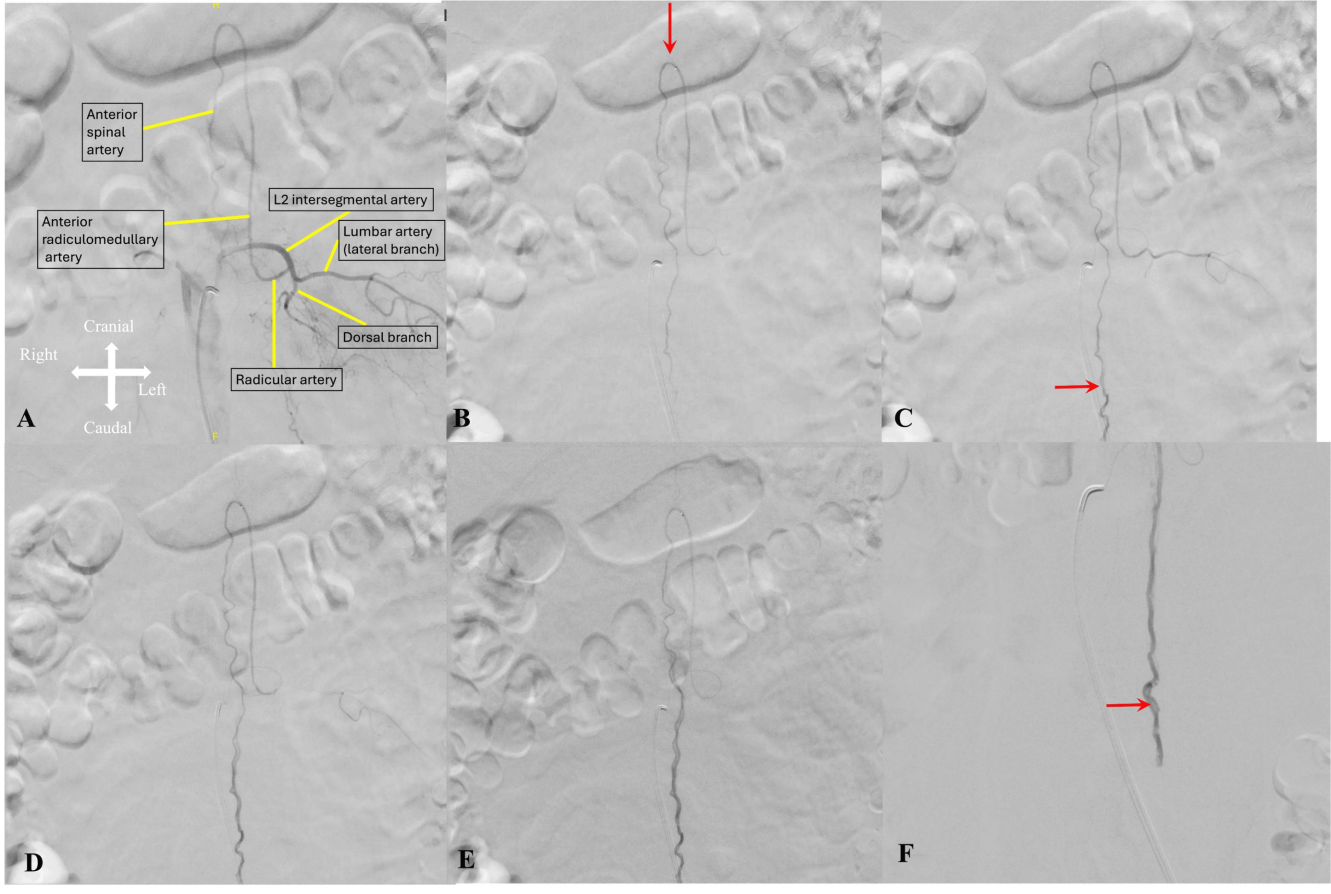
Preoperative digital subtraction angiography. Diagnostic digital subtraction angiography demonstrates the artery of Adamkiewicz arising from the left L2 radicular artery (A), undergoing a hairpin turn supplying the distal anterior spinal artery (B). (A) and (B) show early arterial phases, (C) shows the capillary timing, (D) and (E) show the early and late venous phases, respectively, and (F) shows the delayed venous phase angiogram. The double contour of the draining vein coursing cranially over the feeding artery (red arrow in C) and venous phases (D and E). Also, the stagnant blood flow lingering after the venous phase (red arrow in F) confirms the low-flow nature of the fistula.

A 64-year-old male of African descent presented to the emergency room with lower back pain and bilateral lower extremity weakness of several months’ duration. His only neurological deficit was 4/5 strength in the bilateral lower extremities, most notably proximally in the hip flexors and extensors. An outpatient MRI of the thoracic spine demonstrated cord edema from T7-conus medullaris and multiple flow voids consistent with intradural vessels overlying the spinal cord, which progressed to T1-L2 cord edema on the preoperative MRI scan (Figures 1A-1C). A spinal digital subtraction angiogram (DSA) demonstrated a perimedullary arteriovenous fistula spanning the L2-5 vertebrae supplied by the ASA originating from the artery of Adamkiewicz (Figures 2A-2F).

Angiographic embolization of the lesion under general anesthesia was offered and scheduled. Somatosensory evoked potentials (SSEPs) and motor evoked potentials (MEPs) were monitored for the procedure. A 5-Fr Cobra tip femoral angiography sheath was introduced through the left femoral artery and advanced cranially through the descending aorta under fluoroscopy during the procedure. Contrast dye and overlay mapping were then utilized to identify the artery of Adamkiewicz, which originated at the level of the left L2 intervertebral foramen. Once the AVF was isolated on fluoroscopy, a preembolization trial with lidocaine and pentobarbital greatly diminished SSEPs in the lower extremities, with a similar loss of MEPs. Due to the loss of neuromonitoring signals, it was considered unsafe to proceed with the embolization, and open surgical treatment was planned. In situations where endovascular embolization results in loss of neuromonitoring, open approaches are preferred as occlusion of the feeding artery/arteries can be rapidly reversed by removing the temporary clip to avoid permanent detriment to the spinal cord, which may not be readily resolved during embolization procedures.

**Figure 3:**
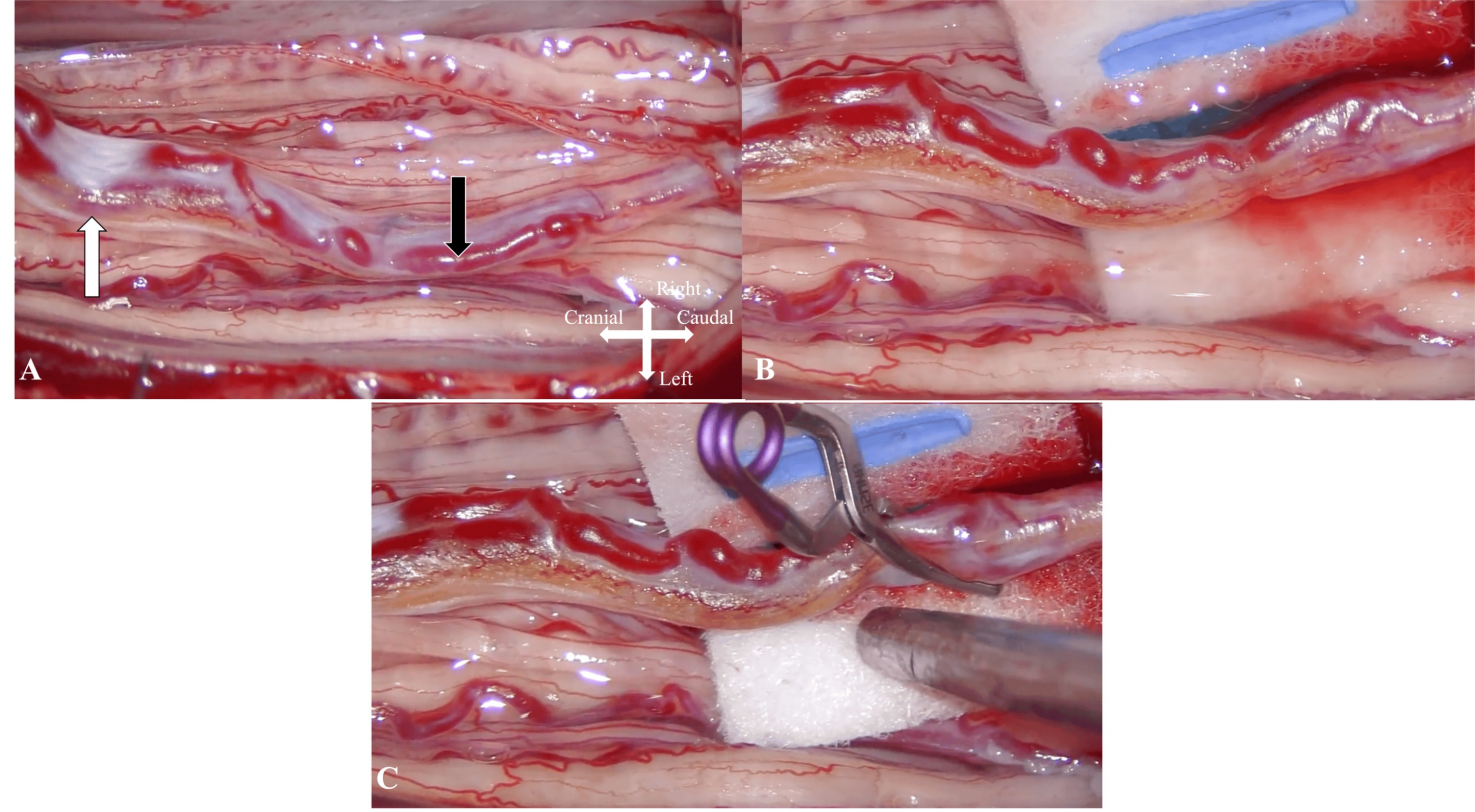
Intraoperative images of arteriovenous fistula visualized utilizing surgical microscope. (A) Arteriovenous fistula exposed following dural opening following L2-4 laminectomy. The engorged arterialized vein (black arrow) over the filum terminale, which looks different from the cauda equina’s nerve bundles and contains fat tissue (white arrow), is noted. (B) Enlarged view of (A) with further isolation of the fistulous filum terminale. (C) An aneurysm clip is placed proximally on the arterialized vein. Following confirmation of obliteration of fistula with intraoperative angiography, the filum terminale containing the fistulous point located just caudal to the aneurysm clip is coagulated and divided.

Following team and patient discussions, microsurgical obliteration of the AVF through an open surgical approach was planned. Following the L2-4 laminectomy, the dura was longitudinally opened under microscopic visualization. After proper extradural hemostasis was achieved, the dura was opened longitudinally and tacked up to the laterally dissected paraspinal musculature. At this point, the cauda equina and filum terminale came into view. A prominent arterialized vein coursing alongside the filum was identified. Indocyanine green (ICG) video angiography confirmed arterialization of the vein at the lower end of L4 with contiguous vessels visualized going caudally and another traveling cephalad. A temporary clip was then applied just cephalad to the site of the AVF, and intraoperative angiography confirmed occlusion of the AVF. No signal change from baseline in neuromonitoring occurred. A permanent clip was then deployed cephalad to the first clip, followed by bipolar cauterization of the filum terminale between the two micro-vascular clips (Figures 3A-3C). The filum terminale was divided, and adequate closure was then achieved in a multilayer fashion. No surgical specimen was sent for pathologic diagnosis.

The patient tolerated the procedure well and his lower extremity weakness was mildly improved compared to presurgical assessment. Postoperative spinal angiography displayed resolution of the FTAVF. Ten days following discharge, while in acute rehab, the patient experienced severe shortness of breath and was diagnosed with a saddle pulmonary embolism. Interventional thrombectomy was attempted and successful. A right lower extremity deep venous thrombosis (DVT) was identified with compression ultrasonography (US). Due to contraindications for antiplatelets and anticoagulants, an inferior vena cava (IVC) filter was placed. The patient was stabilized and discharged to subacute rehabilitation.

## Discussion

Here, we present a case of a 64-year-old male patient presented with myelopathic symptoms of the lower extremities. The patient’s symptoms had quickly progressed from LBP to lower extremity pain and weakness, for which an MRI with and without contrast of the lumbar spine was appropriately performed, demonstrating spinal cord edema from T1-L2. Further investigation revealed a FTAVF at the level of L2-L5, originating from the artery of Adamkiewicz. Endovascular intervention was planned. However, following changes in neuromonitoring during the endovascular approach, the patient underwent successful open surgical intervention.

Spinal AVMs are rare tortuous vascular lesions that often arise in pediatric populations [8]. In 1987, Rosenblum et al. proposed a four-tier classification system for spinal AV shunts [9]. In 1992, Anson and Spetzler further developed the system by adding subclassifications for type IV lesions (Table 1) [4]. The lesion in the present case fits with a type IVa AV shunt [1,5,10]. These lesions are low-flow, high-pressure systems that are often unstable and unpredictable. Due to the low flow in this system, ischemia can occur in the supplied tissue, a condition known as "Foix-Alajouanine syndrome" or "subacute necrotizing myelopathy." This involves progressive congestive ischemia of the spinal cord, which develops over months or years [10]. Progressive myelopathy, radiculopathy, LBP, and bladder or bowel incontinence may also occur during the course of the disease. Due to the high pressure of this system, these lesions are vulnerable to rupture, resulting in hemorrhaging into the subarachnoid space. Rapid, excruciating back pain is often the first symptom, classically referred to as “Coup de poignard of Michon" [10]. Efficient diagnosis and treatment are crucial to avoid catastrophic outcomes in these patients, which may involve permanent damage to the spinal cord and possibly death.

**Table 1:** Classification of spinal cord arteriovenous malformations. The classification table is developed with the help of the studies by Rosenblum et al. [9], Anson and Spetzler [4], and Spetzler et al. [11]. AVF: arteriovenous fistula; aa.: arteries; ASA: anterior spinal artery; PSA: posterior spinal artery

Type	Subtype	AV shunt	Characteristics	Typical feeding vessel(s)
I	N/A	Dural AVF	Vascular nidus embedded within the dura mater	Intercostal aa., lumbar aa.
II	N/A	Intramedullary AVM	Vascular nidus embedded within pia mater or spinal cord parenchyma	Medullary aa.
III	N/A	Intramedullary juvenile AVF	Vascular nidus embedded within the pia mater, spinal cord parenchyma present within the nidus	Medullary aa.
IV	IVa	Perimedullary AVF	Small with a single feeder	ASA
	IVb		Medium with multiple feeders	ASA, PSA
	IVc		Giant with multiple feeders	ASA, PSA
V	Va	Extradural AVF	Epidural AVF with intradural venous drainage	Radicular aa.
	Vb		Epidural AVF without intradural venous drainage	Radicular aa.

In cases of FTAVFs, the main cause of neurological symptoms is unlikely to be due to direct ischemia or compression of the AVF on the FT or adjacent nerve roots. The cauda equina typically has adequate space to maneuver and the FT rarely carries any meaningful neurologic signals. The symptoms are thought to be primarily due to the venous congestion caused by the AVF, affecting the levels of cephalad to the fistula, and can cause myelopathic or radicular symptoms [6,7,12]. On MRI, venous congestion is visualized in the form of spinal cord edema at the spinal levels, where congestion has impacted normal vascular dynamics [13]. Importantly, this edema, and presumably the venous stasis, is typically improved or eliminated when FTAVFs are promptly treated [12]. Many patients affected by FTAVFs also present with lumbar spinal stenosis, leading some to hypothesize that longstanding neural compression and inflammation can contribute to AVF formation [6,14]. In the cases indexed in this literature review, 17 cases reported the presence of lumbar spinal stenosis (nine cases reported the absence of lumbar spinal stenosis and 32 cases failed to report the absence or presence of stenosis). The presence of concurrent lumbar stenosis has the potential to mask the true cause of symptoms, especially when symptoms are primarily radicular, causing a delay in diagnosis.

Treatment for FTAVFs may include surgical, endovascular, or radiotherapeutic management. The surgical approach has previously been established as the modality of choice, with the first successful treatment in 1916 [15]. This approach involves occlusion of the receiving vein of the shunt, with definitive interruption of other spinal draining veins. This is crucial for successful treatment, as occlusion of arterial feeders may result in re-establishment of the fistula via recruitment of new arterial feeders, which can lead to relapsing symptoms [16]. Surgical management has been shown to be the most definitive treatment [17,18]. However, endovascular treatment has recently seen a surge in popularity in treating spinal AVFs [19]. Many institutions utilize endovascular techniques as first-line treatment as it is less invasive. While no difference has been seen when comparing complication rates between surgical and endovascular management for spinal AVFs, embolization is associated with a much higher failure rate, with patients often having to return for open surgery or repeat endovascular embolization [18,20-23]. Finally, stereotactic radiosurgery has also been described in the literature as a means to treat dural AVFs [24]. However, it has not been established as a mode of treatment for perimedullary AVFs, and with the availability of other effective treatment options, radiosurgery is currently not recommended as a management option in most AVF cases [25].

We indexed and reviewed 24 articles with 59 cases in the literature that reported FTAVFs with either progressive myelopathy and/or radiculopathy. The identified feeding vessel(s) and subsequent draining vein(s), chosen treatment options, complications, and outcomes are shown in Table 2. The patients' ages ranged from 3 to 84 years, with 38 males, nine females, and two unidentified. FTAVFs were more common in males, which is consistent with previously published literature [26]. We compared the approach of treating AVFs (for which both endovascular and microsurgical approaches have been frequently utilized) by observing outcomes and intraoperative or postoperative complications. Both approaches offered positive outcomes, resulting in improvement, if not resolution of symptoms in a majority of cases. However, in previously reported cases of FTAVF, endovascular treatment was associated with more complications (46.7%), with failed embolization being the reported complication in all cases, requiring repeat embolization or subsequent microsurgical intervention. There were two cases of microsurgical complications in which patients experienced worsening urinary symptoms. Cases treated with microsurgery reported higher success rates with complete resolution being identified in 14 of the 59 cases. Compared to endovascular approaches which had no cases reporting complete symptom resolution. Finally, microsurgical management reported two cases where symptoms were unchanged, compared to one case that was approached endovascularly.

**Table 2:** Selected previously reported cases describing filum terminale arteriovenous fistulas. M: male; a of FT: artery of filum terminale; v of FT: vein of filum terminale; NS: not stated; ASA: anterior spinal artery; Y: yes; LSA: lateral sacral artery; N: no; a of adam: artery of Adamkiewicz; F: female; SRA: segmental radicular artery

Authors	Age/sex	Type	Presentation	Feeding a	Draining v	LSS	Treatment	Complications	Outcome
Meisel et al. (1995) [27]	30/M	IVa	Radiculopathy	a of FT	v of FT	NS	Microsurgery	NS	Resolved
Tender et al. (2005) [28]	70/M	IVa	Myelopathy	ASA	v of FT	Y	Microsurgery	NS	Resolved
	58/M	IVa	Myelopathy	ASA	v of FT	Y	Microsurgery	NS	Resolved
Jin et al. (2010) [29]	61/M	IVb	Polyradiculopathy	LSA	Venous plexus	NS	Microsurgery, endovascular	Failed embolization	Resolved
Trinh and Duckworth 2011) [14]	57/M	IVa	Polyradiculopathy	ASA	v of FT	Y	Microsurgery	Cautery injury S3-4 nerve roots, impaired bladder/bowel control, pseudo-meningocele	Improved
	63/M	IVb	Polyradiculopathy	NS	Venous plexus	Y	Microsurgery	None	Improved
Witiw et al. (2011) [30]	62/M	IVa	Polyradiculopathy	ASA	v of FT	N	Microsurgery	None	Improved
Kumar et al. (2011) [31]	44/M	IVa	Myeloradiculopathy	a of adam	v of FT	Y	Microsurgery	None	Resolved
Lim et al. (2011) [32]	60/M	IVa	Myeloradiculopathy	ASA	v of FT	N	Microsurgery	None	Improved
	48/M	IVa	Myeloradiculopathy	ASA	v of FT	N	Microsurgery	None	Improved
	53/F	IVc	Myeloradiculopathy	Multiple ASA branches	v of FT	N	Endovascular	None	Improved
	63/F	IVb	Myeloradiculopathy	ASA, LSA	Venous plexus	N	Endovascular	2 staged embolization	Improved
Macht et al. (2011) [33]	57/M	IVb	Myeloradiculopathy	ASA, SRA	v of FT	N	Endovascular	None	Improved
Takami et al. (2012) [34]	66/F	IVa	Polyradiculopathy	ASA	v of FT	Y	Microsurgery	Worsening urinary ssx	Improved
	63/M	IVa	Polyradiculopathy	ASA	v of FT	Y	Microsurgery	None	Improved
Chanthanaphak et al. (2013) [13]	70/F	IVa	Myeloradiculopathy	ASA	v of FT	Y	Endovascular	None	Improved
	55/M	IVa	Myeloradiculopathy	ASA	v of FT	NS	Endovascular	None	Resolved
	63/M	IVa	Myeloradiculopathy	ASA	v of FT	NS	Microsurgery	None	Improved
	39/F	IVa	Myeloradiculopathy	ASA	v of FT	NS	Endovascular	None	Improved
	31/M	IVa	Myeloradiculopathy	ASA	v of FT	NS	Endovascular	None	Improved
	67/M	IVa	Myeloradiculopathy	ASA	v of FT	NS	Endovascular	None	Improved
	72/M	IVa	Myeloradiculopathy	ASA	v of FT	NS	Endovascular	None	Improved
	57/F	IVa	Myeloradiculopathy	ASA	v of FT	NS	Microsurgery	None	Improved
	66/M	IVa	Myeloradiculopathy	ASA	v of FT	NS	Microsurgery, Endovascular	Failed embolization	Improved
	62/M	IVa	Polyradiculopathy	ASA	v of FT	NS	Microsurgery	None	Improved
Krishnan et al. (2013) [35]	54/M	IVa	Myeloradiculopathy	ASA	v of FT	Y	Microsurgery	None	Resolved
Sharma et al. (2014) [36]	48/M	IVa	Myeloradiculopathy	ASA, a of FT	v of FT	Y	Microsurgery	None	Resolved
Ding et al. (2016) [37]	43/M	IVa	Myeloradiculopathy	ASA	v of FT	Y	Microsurgery	None	Resolved
Hong et al. (2017) [2]	52.9 +/- 12.6, 8M, 3F	IVa	Myeloradiculopathy	a of FT	v of FT	NS	Microsurgery	NS	Improved
		IVa	Myeloradiculopathy	a of FT	v of FT	NS	Microsurgery	NS	Improved
		IVa	Myeloradiculopathy	a of FT	v of FT	NS	Microsurgery	NS	Improved
		IVa	Myeloradiculopathy	a of FT	v of FT	NS	Microsurgery	NS	Improved
		IVa	Myeloradiculopathy	a of FT	v of FT	NS	Microsurgery	NS	Improved
		IVa	Myeloradiculopathy	a of FT	v of FT	NS	Microsurgery	NS	Improved
		IVa	Myeloradiculopathy	a of FT	v of FT	NS	Microsurgery	NS	Improved
		IVa	Myeloradiculopathy	a of FT	v of FT	NS	Microsurgery	NS	Improved
		IVb	Myeloradiculopathy	a of FT, LSA	v of FT	NS	Microsurgery	NS	Improved
		IVb	Myeloradiculopathy	a of FT, LSA	v of FT	NS	Microsurgery, Endovascular	Failed embolization	Improved
		IVb	Myeloradiculopathy	a of FT, MSA	v of FT	NS	Microsurgery, Endovascular	Failed embolization	Improved
		IVc	Myeloradiculopathy	a of FT	v of FT	NS	Microsurgery	NS	Improved
Wajima et al. (2017) [38]	78/M	IVb	Myeloradiculopathy	LSA	v of FT	N	Endovascular	Repeat endovascular treatment	Improved
Hong et al. (2018) [39]	45/M	IVb	Myeloradiculopathy	a of FT	v of FT	NS	Microsurgery	None	Improved
	31/M	IVb	Myeloradiculopathy	a of FT	v of FT	NS	Microsurgery	None	Improved
Lee et al. (2019) [40]	53/M	IVa	Myeloradiculopathy	ASA	v of FT	N	Microsurgery	None	Resolved
Takai et al. (2019) [41]	73/M	IVa	Myeloradiculopathy	ASA	v of FT	NS	Microsurgery	None	Unchanged
	63/F	IVa	Myeloradiculopathy	ASA	v of FT	NS	Microsurgery	None	Improved
	76/M	IVa	Myeloradiculopathy	ASA	v of FT	NS	Microsurgery	None	Improved
	84/M	IVa	Myeloradiculopathy	a of FT	v of FT	NS	Microsurgery	None	Unchanged
	83/M	IVa	Myeloradiculopathy	ASA	v of FT	NS	Microsurgery	None	Improved
	54/M	IVa	Myeloradiculopathy	ASA	v of FT	NS	Microsurgery	None	Improved
	40/M	IVa	Myeloradiculopathy	ASA	v of FT	NS	Microsurgery	None	Unchanged
Scullen et al. (2019) [6]	62/M	IVa	Myeloradiculopathy	ASA	v of FT	Y	Microsurgery	None	Resolved
Lakhdar et al. (2019) [42]	43/M	IVa	Myeloradiculopathy	ASA	v of FT	NS	Microsurgery	None	Resolved
Iampreechakul et al. (2020) [1]	58/M	IVb	Myeloradiculopathy	ASA, LSA	v of FT	Y	Microsurgery	None	Resolved
	70/M	IVb	Myeloradiculopathy	ASA, LSA	v of FT	Y	Endovascular	Failed embolization, pt refused microsurgery	Unchanged
	52/F	IVb	Myeloradiculopathy	LSAx2	v of FT	Y	Microsurgery, Endovascular	None	Resolved
Farinha et al. (2021) [43]	73/M	Iva	Myeloradiculopathy	ASA	v of FT	Y	Microsurgery	None	Improved
Iampreechakul et al. (2021) [44]	64/M	Iva	Myelopathy	a of FT	v of FT	Y	Microsurgery	None	Resolved
El Naamani et al. (2022) [45]	62	Iva	Radiculopathy	a of adam	v of FT	N	Microsurgery	None	Improved

## Conclusions

This case demonstrates the importance of early identification and treatment of AVFs, as well as the importance of a multidisciplinary therapeutic approach. In this case, endovascular embolization was attempted; however, it was aborted due to loss of neuromonitoring signal, and open surgical management was scheduled. Successful treatment was achieved with microsurgery, with improvement immediately postoperatively. While endovascular management is often highly successful in treating FTAVFs, surgeons should be prepared for microsurgical treatment if embolization fails or is unsafe to proceed.
